# Validation of the EYEMATE‐SC Suprachoroidal Pressure Transducer for Telemetric Measurement of Intraocular Pressure in Normal Ex Vivo Canine and Equine Globes—Preliminary Results

**DOI:** 10.1111/vop.70071

**Published:** 2025-08-29

**Authors:** Phillip N. Buckman, Bailey A. Brinker, Lydia E. Kapeller, András M. Komáromy

**Affiliations:** ^1^ Department of Small Animal Clinical Sciences College of Veterinary Medicine, Michigan State University East Lansing Michigan USA

**Keywords:** dog, glaucoma, horse, intraocular pressure sensor, manometry, ultrasound biomicroscopy (UBM)

## Abstract

**Objective:**

To determine the accuracy of the EYEMATE‐SC suprachoroidal tracer for telemetric tonometry in canine and equine globes.

**Procedures:**

The EYEMATE‐SC sensor (7.8 mm × 3.8 mm × 1 mm) was implanted in the suprachoroidal space of four freshly enucleated normal canine and two normal equine eyes. The anterior chambers were cannulated and connected to a reservoir of Plasma‐Lyte A and a manometer. Starting at a manometric IOP of 5 mmHg, the pressure was progressively increased to 80 mmHg by raising the reservoir. At each setpoint (5, 10, 15, 20, 25, 30, 35, 40, 50, 60, 70, and 80 mmHg), triplicate telemetric measurements were taken with the EYEMATE‐SC using a portable reading device for telemetric pressure transmission via a radiofrequency band. These measurements were compared to manometric pressure by linear regression analysis.

**Results:**

A strong positive linear regression was observed between EYEMATE‐SC and manometry IOPs in both canine and equine eyes (canine: *R* ^2^ = 0.99; equine: *R* ^2^ = 0.99). The EYEMATE‐SC was unable to measure pressures > 70 mmHg in either species.

**Conclusions:**

Measuring canine and equine IOPs from the suprachoroidal space using the EYEMATE‐SC provided accurate results over an extensive range of pressures in ex vivo globes. This telemetric sensor could assist with long‐term, frequent tonometry by pet owners and clinicians following in vivo testing. Although the sensor could not detect pressures above 70 mmHg, this flaw was not considered clinically relevant.

## 1 Introduction

Glaucoma is a group of progressive optic neuropathies characterized by the loss of retinal ganglion cells, resulting in optic nerve degeneration and optic nerve head cupping [1, 2, 3]. Intraocular pressure (IOP)‐related biomechanical stress is a major risk factor [1, 2, 3]. Glaucoma is a leading cause of vision loss in humans and animals [1, 2, 3]. Depending on the presence of an identifiable underlying cause, glaucoma is classified as either primary or secondary [1, 2, 3]. While primary glaucoma is common in humans and dogs, secondary glaucomas are the dominant disease form in other species, such as cats and horses. They are often caused by chronic and recurrent uveitis [1, 2, 3, 4, 5]. Unless an underlying cause can be identified and addressed, there is no cure for glaucoma, and the treatment is limited to IOP lowering by medical and surgical means [1, 2, 3].

The detection of elevated IOP by applanation or rebound tonometry is an essential part of glaucoma diagnostics in humans and animals [1, 2, 3, 4, 5]. These measurements are generally limited to office visits and only provide a snapshot of an individual's IOP. Continuous, telemetric IOP measurements are expected to provide a more accurate assessment of true IOP in the future. Several systems are being developed, but availability for clinical application is limited [6]. A rebound tonometer is being marketed for patients to measure their IOP at home, and some veterinary ophthalmologists are providing pet owners with rebound tonometers for use at home [7, 8]. While home tonometry does not provide continuous IOP measurements, it does offer additional valuable data points between office visits.

**FIGURE 1:**
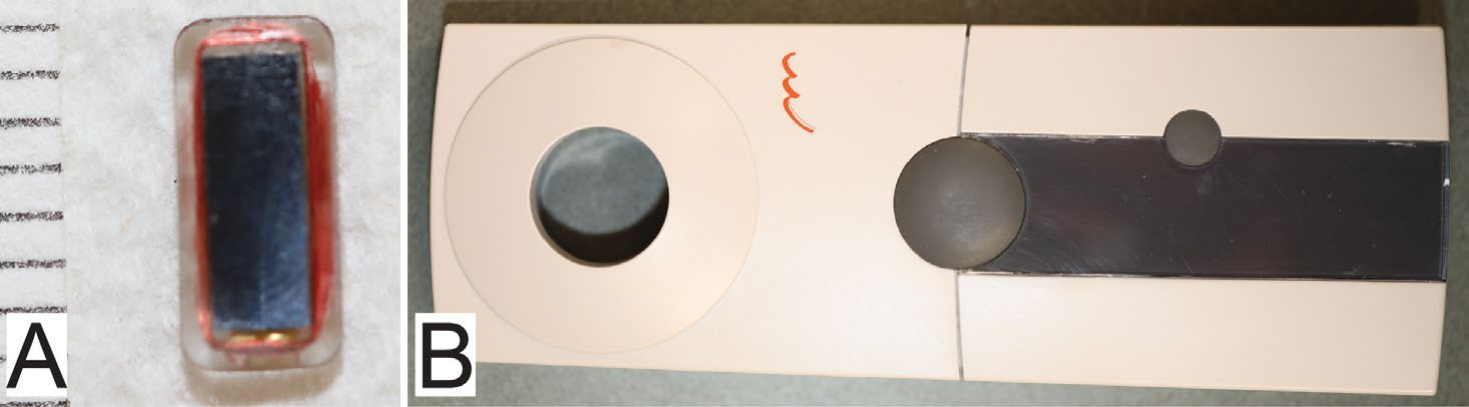
The EYEMATE‐SC. The implantable suprachoroidal pressure transducer (7.8 mm × 3.8 mm × 1 mm) is embedded in a silicone rubber encasement (A). The ruler on the left shows a millimeter scale (1 mm between two lines). The portable reading device serves for the telemetric pressure transmission via a radio frequency band (B).

In 2021, the EYEMATE‐SC pressure transducer (Implandata Ophthalmic Products GmbH) was approved for clinical use in humans in Europe (Figure 1A) [9, 10, 11]. The EYEMATE‐SC is a micro‐electro‐mechanical system application‐specific integrated circuit (MEMS ASIC) comprising pressure‐sensitive sensor cells, temperature sensors, analog‐to‐digital converters, and telemetry [12, 13]. The ASIC is bonded to a gold‐made circular micro‐coil antenna [12, 13]. Both parts are silicon encapsulated [12, 13]. This device is placed surgically in the suprachoroidal space and allows the measurement of IOP with a portable reading device for telemetric pressure transmission via a radiofrequency band (Figure 1B) [9, 10, 11, 14]. The safety and accuracy of the EYEMATE‐SC have been demonstrated in rabbits over 30 weeks and in human patients with open‐angle glaucoma over three years [9, 10, 11, 14]. The purpose of our ex vivo study was to determine the accuracy of the EYEMATE‐SC for telemetric tonometry in canine and equine globes.

## 2 Methods

### 2.1 Animals

No live animals were used, and no animals were sacrificed for the sole purpose of this study. Our work adhered to the Association for Research in Vision and Ophthalmology (ARVO) Statement for the Use of Animals in Ophthalmic and Vision Research and was approved by the Charles Rivers Laboratories Institutional Animal Care and Use Committees (IACUC).

Four freshly enucleated normal eyes were obtained from four adult female laboratory Beagles, between two and three years old. These eyes were provided by a local contract laboratory (Charles Rivers Laboratories) and collected postmortem from dogs euthanized for another unrelated study. No live dogs were used or euthanized specifically for this study. The eyes were shipped overnight on ice, and the studies were performed within 24 h of collection. Two equine eyes were collected from a 16‐year‐old female Quarter horse euthanized for health reasons at the Michigan State University Veterinary Medical Center. The owner provided written consent for the use of the eyes, which were used within 24 h of collection.

The cadaver eyes were examined by a board‐certified veterinary ophthalmologist (AMK) for abnormalities by slit‐lamp biomicroscopy (Kowa SL17; Kowa Company) and binocular indirect ophthalmoscopy (Keeler All Pupil II; Keeler Instruments) with condensing lens (Double Aspheric Pan Retinal 2.2D; Volk Optical). Except for a few iris‐to‐iris persistent pupillary membrane strands in the two equine eyes, no other abnormalities were observed.

### 2.2 Surgical Placement of the EYEMATE‐SC

**FIGURE 2:**
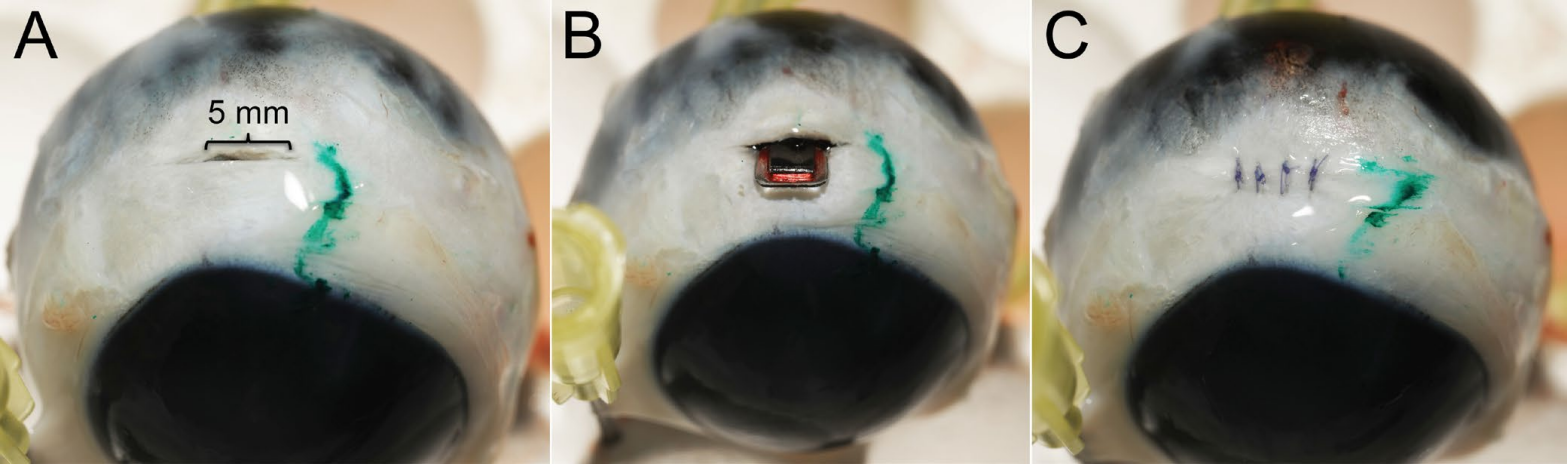
Suprachoroidal implantation of the EYEMATE‐SC sensor device in a canine globe. (A) A small, 5‐mm incision was made over the pars plana of the ciliary body parallel to the limbus. (B) Following the creation of a suprachoroidal pocket with viscoelastic solution, the EYEMATE‐SC was inserted through the scleral incision, perpendicular to the limbus. (C) The scleral incision was closed with a simple interrupted suture pattern using 8–0 polyglactin 910.

We followed the surgical techniques previously described for rabbits and humans to place the EYEMATE‐SC into the suprachoroidal space of the canine and equine eyes [9, 14]. A dissecting microscope was used for the surgical procedure (Nikon SMZ645 Stereo Microscope). The conjunctiva and Tenon's capsule were removed over three clock hours to expose the superior temporal sclera. A small, 5‐mm incision was made with a 6400 Beaver blade (Surgical Specialties Mexico) over the pars plana of the ciliary body parallel to the limbus (Figure 2A). The distance of the scleral incision from the limbus was 6 mm for the dog and 10 mm for the horse [15, 16]. A pocket was created distal from the scleral incision by injecting 1.8% sodium hyaluronate viscoelastic (an‐bfh 1.8%; An‐Vision Inc.) through a 27G blunt‐tipped cannula into the suprachoroidal space [9, 14]. Using the tying platform of non‐toothed Castroviejo tying forceps, the EYEMATE‐SC transducer was carefully inserted into the suprachoroidal pocket, perpendicular to the limbus (Figure 2B). The scleral incision was closed with a simple interrupted suture pattern using 8–0 polyglactin 910 (8–0 Coated Vicryl; Ethicon LLC) to provide a watertight seal (Figure 2C).

### 2.3 Manometry

EYEMATE‐SC IOP readings were compared to direct manometry as described before [17, 18]. In brief, the eyes were positioned with the corneal surface perpendicular to a horizontal surface. The anterior chamber was cannulated through the corneal limbus at the 3 and 9 o'clock positions using two 25‐gauge 5/8‐in. needles. One of the needles was attached to a 1‐L bag of Plasma‐Lyte A injection fluid (PLASMA‐LYTE A Injection pH 7.4, Baxter Healthcare Corporation), and the other needle was attached to a calibrated manometer (Traceable Manometer Model 3460; Control Company) with plastic tubing provided by the manufacturer [17, 18]. No leakage was observed at any cannulation site. The manometric IOP was increased in 5‐mmHg increments from 5 to 40 mmHg and then in 10‐mmHg increments to 80 mmHg by raising the fluid bag.

At each setpoint (5, 10, 15, 20, 25, 30, 35, 40, 50, 60, 70, and 80 mmHg), triplicate telemetric measurements were taken by the same individual (PNB) by holding the circle‐shaped opening of the portable reading device (Figure 1B) over the transducer implantation site. These EYEMATE‐SC measurements were compared to the direct manometric pressure by linear regression analysis (Microsoft Excel).

### 2.4 Ultrasound Biomicroscopy (UBM)

Following the manometric measurements, the location of the EYEMATE‐SC transducer in the suprachoroidal space was confirmed in the two equine eyes by UBM using a 48‐MHz probe (UBM Plus; Accutome Inc.). Unfortunately, UBM was not available when the canine eyes were tested.

## 3 Results

### 3.1 EYEMATE‐SC Provides Accurate IOP Readings

**FIGURE 3:**
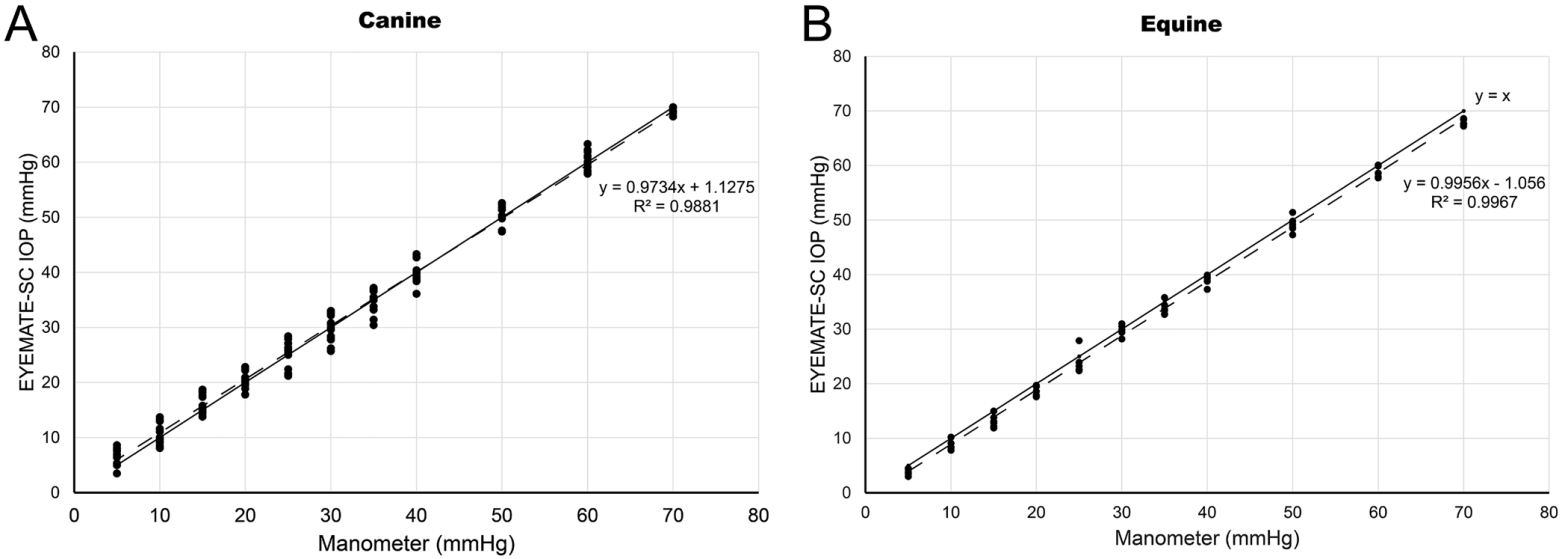
Comparison between direct manometry and telemetric measured IOP. Linear regression analysis was performed to compare the EYEMATE‐SC (*y*‐axis) to direct manometry (*x*‐axis) in canine (A) and equine (B) eyes. Linear equations and coefficients of determination (*R* ^2^) are included in the scatter plots. There was an almost perfect agreement between the dashed regression lines and the solid *y* = *x* line.

A strong positive linear regression was observed between EYEMATE‐SC and manometry IOPs in both canine and equine eyes (canine: *R* ^2^ = 0.99; equine: *R* ^2^ = 0.99; Figure 3). The EYEMATE‐SC was unable to measure pressures > 70 mmHg in either species.

### 3.2 UBM Confirms the Location of the EYEMATE‐SC in the Suprachoroidal Space

**FIGURE 4:**
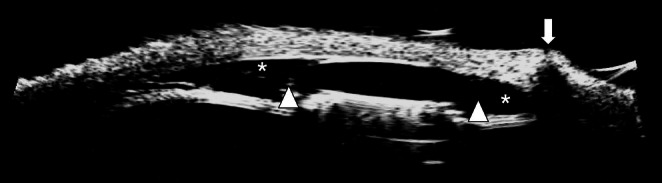
Longitudinal scan using UBM to visualize the EYEMATE‐SC. The transducer (between the two arrowheads) is located in the suprachoroidal space (*), which is locally expanded with viscoelastic solution. The vertical arrow shows the scleral incision through which the implant was inserted into the suprachoroidal space.

Ultrasound biomicroscopy was performed in the two equine eyes to confirm the location of the EYEMATE‐SC transducers in the suprachoroidal space (Figure 4). The suprachoroidal space appeared expanded due to the injected viscoelastic solution.

## 4 Discussion

Measuring canine and equine IOPs from the suprachoroidal space using the EYEMATE‐SC provided accurate results over an extensive range of pressures in ex vivo canine and equine globes. Our data confirm previous results published in rabbits, where IOPs measured with the EYEMATE‐SC were compared to direct manometry in vivo over 30 weeks post suprachoroidal implantation [14]. The average difference between telemetric and manometric IOP was 0.31 mmHg across all sensors and time points, with a coefficient of determination (*R* ^2^) of 0.99, similar to that in the current study [14]. Even though direct manometry was not performed, clinical measurements showed excellent agreement between the EYEMATE‐SC transducer pressure readings and Goldmann applanation tonometry in human patients with open‐angle glaucoma over more than three years [9, 10, 11]. EYEMATE‐SC IOPs were on average within 0.8 to 2.3mmHg of Goldmann applanation tonometry readings [9, 10, 11]. The EYEMATE‐SC was approved in Europe for clinical use in human patients in June 2021 [9, 10, 11].

The excellent agreement with direct manometry is understandable, as the EYEMATE‐SC also provides direct IOP measurements. This means that the readings are not affected by abnormalities of the cornea, which affect indirect measurements, such as applanation and rebound tonometry [19].

Ophthalmologic examinations did not show any adverse effects from the EYEMATE‐SC in humans and rabbits [9, 10, 11, 14]. Histologic analyses in rabbits revealed a small band of fibrosis adjacent to the implantation site 30 weeks after sensor placement [14]. There were no signs of other pathologies, such as inflammation or necrosis [14].

The next step in our investigations will be the clinical application of the EYEMATE‐SC in clinical animal patients. The ability to measure IOPs between office visits with veterinary ophthalmologists is crucial in improving our ability to diagnose glaucoma and monitor responses to therapy [7, 8]. Home rebound tonometry is already being performed by human patients and is being prescribed by some veterinary ophthalmologists [7, 8]. To the best of our knowledge, there are no published data on home tonometry in animals. The EYEMATE‐SC provides another tool for home tonometry by animal owners. While pet owners may not agree to perform surgery for the sole purpose of transducer implantation, the device could be easily combined with other ocular surgeries, as shown in human patients, where the EYEMATE‐SC was implanted in human open‐angle glaucoma patients who underwent simultaneous non‐penetrating glaucoma surgeries, such as canaloplasty or deep sclerectomy [9]. In animals, the EYEMATE‐SC could be implanted in combination with canine and equine cataract surgery or following tube shunt placement in dogs. The suprachoroidal space is routinely accessed for the placement of cyclosporine A drug implants in horses with equine recurrent uveitis [20]. We anticipate that the suprachoroidal space could be easily expanded by viscoelastic dissection to insert the EYEMATE‐SC.

The inability of the EYEMATE‐SC to measure IOPs over 70 mmHg is not a major limitation. Animal owners should be advised to contact their veterinary ophthalmologist if they are unable to obtain a reading with the EYEMATE‐SC.

While the EYEMATE‐SC can assist with long‐term, frequent tonometry by pet owners and clinicians, continuous IOP readings are not possible with the EYEMATE‐SC. However, when a similar implant (EYEMATE‐IO) was placed intraocularly in the ciliary sulcus, continuous tonometry was performed short‐term via a custom‐built antenna placed on the skin around the eye in human glaucoma patients, suggesting that continuous tonometry may be possible with the EYEMATE‐SC, at least short‐term [13, 21, 22].

Limitations of this study include the small sample size and the lack of canine UBM images. However, given the robust safety, efficacy, and imaging data available from previous studies in rabbits and humans [9, 10, 11, 14], current findings provide a strong basis for proceeding to in vivo evaluation in canine and equine patients.

In conclusion, the EYEMATE‐SC represents a potential option for home tonometry, an alternative to rebound tonometry. Extensive efficacy and safety data are available for rabbits and humans [9, 10, 11, 14], and our study provides efficacy data for canine and equine eyes. Further in vivo research is required in these two species to determine the usefulness of the EYEMATE‐SC for effective long‐term use.

## Ethics Statement

This study adhered to the Association for Research in Vision and Ophthalmology (ARVO) Statement for the Use of Animals in Ophthalmic and Vision Research. The Charles Rivers Laboratories Institutional Animal Care and Use Committee (IACUC) approved the collection of donated canine tissues. The horse owner provided written consent for the use of the equine eyes following euthanasia. No live animals were used, and no animals were sacrificed for the sole purpose of this study.

## Conflicts of Interest

A.M.K. received research funding from PolyActiva Pty. Ltd., AbbVie Inc., and W. L. Gore & Associates Inc. while the presented work was conducted. While A.M.K. also serves as Editor‐in‐Chief of Veterinary Ophthalmology, he was not involved in the review of this manuscript. All other authors declare no conflicts of interest.

## Data Availability

The data that support the findings of this study are available from the corresponding author upon reasonable request.
